# Explorations of object and location memory using fMRI

**DOI:** 10.3389/fnbeh.2013.00105

**Published:** 2013-08-15

**Authors:** Antony D. Passaro, L. Caitlin Elmore, Timothy M. Ellmore, Kenneth J. Leising, Andrew C. Papanicolaou, Anthony A. Wright

**Affiliations:** ^1^DCS CorporationAlexandria, VA, USA; ^2^Department of Neuroscience, Baylor College of MedicineHouston, TX, USA; ^3^Department of Psychology, The City College of New YorkNew York, NY, USA; ^4^Department of Psychology, Texas Christian UniversityFort Worth, TX, USA; ^5^Department of Pediatrics, The University of Tennessee Health Science CenterMemphis, TN, USA; ^6^Department of Neurobiology and Anatomy, The University of Texas Health Science CenterHouston, TX, USA

**Keywords:** fMRI, working memory, location, object, domain specificity

## Abstract

Content-specific sub-systems of visual working memory (VWM) have been explored in many neuroimaging studies with inconsistent findings and procedures across experiments. The present study employed functional magnetic resonance imaging (fMRI) and a change detection task using a high number of trials and matched stimulus displays across object and location change (what vs. where) conditions. Furthermore, individual task periods were studied independently across conditions to identify differences corresponding to each task period. Importantly, this combination of task controls has not previously been described in the fMRI literature. Composite results revealed differential frontoparietal activation during each task period. A separation of object and location conditions yielded a distributed system of dorsal and ventral streams during the encoding of information corresponding to bilateral inferior parietal lobule (IPL) and lingual gyrus activation, respectively. Differential activity was also shown during the maintenance of information in middle frontal structures bilaterally for objects and the right IPL and left insula for locations. Together, these results reflect a domain-specific dissociation spanning several cortices and task periods. Furthermore, differential activations suggest a general caudal-rostral separation corresponding to object and location memory, respectively.

## Introduction

The ability to perceive and store visual information for a short period of time is essential for daily goal-directed tasks. In combination with the mental manipulation of visual information, this process is often referred to as visual working memory (VWM) (Baddeley, 1986). Neuroimaging studies on VWM have primarily focused on identifying active brain regions during the encoding and maintenance of visual information. Consequently, a functional network of brain regions in the lateral prefrontal and parietal cortices has been described in a variety of VWM tasks (Cohen et al., 1997; Courtney et al., 1997; D'Esposito et al., 1998b; Smith and Jonides, 1998; Postle and D'Esposito, 1999b; Cabeza and Nyberg, 2000; Haxby et al., 2000; Postle et al., 2000; Munk et al., 2002; Pessoa et al., 2002; Linden et al., 2003; Mottaghy et al., 2003; Sala et al., 2003; Sala and Courtney, 2007). Several functional magnetic resonance imaging (fMRI) studies published toward the end of 20th century focused primarily on identifying a domain-based segregation of working memory within the prefrontal cortex for objects and locations (Courtney et al., 1996, 1997, 1998; McCarthy et al., 1996; Belger et al., 1998; Kelley et al., 1998; Smith and Jonides, 1999). However, other fMRI studies, also published during this time, reported results contradictory to domain-based segregation (McCarthy et al., 1994; D'Esposito et al., 1998a; Owen et al., 1998; Petit et al., 1998; Postle and D'Esposito, 1999a,b). In the period that followed, investigators began shifting their focus to other brain regions in an attempt to elucidate what domain-specificity (object vs. location) might exist within working memory. Among these regions were the medial temporal lobe (e.g., hippocampal gyrus), parietal lobe [e.g., intraparietal sulcus (IPS)], and occipital lobe (e.g., lateral-occipital complex) (Ranganath et al., 2004; Buffalo et al., 2006; Bellgowan et al., 2009; Harrison et al., 2010; Cichy et al., 2011). Moreover, several studies reported domain-specific differences between object and location memory that extended across the entire brain (Sala et al., 2003; Mohr et al., 2006; Sala and Courtney, 2007). Overall, these studies supported the theory that during maintenance, visual information is segregated anatomically in the brain depending on the nature of the information, be it a color, a location, or an orientation. This theory of information segregation during maintenance may appear inefficient as it requires the brain to dissect visual information in terms of physical attributes and store them accordingly. However, we know from early studies with nonhuman primates (Ungerleider and Mishkin, 1982) that the brain naturally segments visual objects in terms of their location and identity in the occipital lobe and extends into the parietal and temporal cortices, respectively. Accordingly, the most efficient approach would involve the maintenance of each of these components separately rather than combining components to form the complete mental representation of the encoded item. While an information-segregated or “domain-specific” process appears to be the most efficient means by which to maintain visual information, there is a lack of agreement in the neuroimaging literature to support such a model. Advanced imaging techniques and the development of more comprehensive working memory tasks should allow for a more cohesive framework from which to develop a model of VWM domain-specificity.

Many fMRI studies which focus on segregating domain-specific working memory have used a separate set of parameters, stimuli, and/or task demands across conditions. Furthermore, some studies use a small number of display items (one to three) which allow subjects to approach ceiling performance, and consequently, the load on processing may not be sufficient to create robust differences between VWM domains. In order to identify neural components of memory as unique to a particular condition, it is imperative that the conditions remain as similar as possible for several reasons. If the stimuli or the task parameters (timing, number of items, location of items, etc.) vary across conditions then the underlying neural correlates associated with each condition and thus each domain of VWM may in fact relate more to a specific component of the condition itself, which is not necessary for remembering an identity or a location. Additionally, if performance varies from one condition to the next, any observed difference in brain activity may be due to the increased (or possibly decreased) demand, complexity, or time required to successfully complete the task condition.

The present study used a change detection task with identical displays across paired conditions such that the types of change (location or color) were counter-balanced by using identical stimulus displays. The change-detection task in this study enabled similar performance across object and location conditions while maintaining performance below ceiling. The subsequent data analyses were applied without a predefined notion of anatomically segregated domain specificity. Furthermore, the purpose of this study was to explore the theory that location and identity brain patterns vary across the entire brain when controlling for procedural parameters, such as visual displays and a large number of trials. Contrary to the early view of domain-based segregation in frontal lobe regions, we hypothesize that no such dissociation within the frontal regions will be observable during the maintenance period when identical stimulus displays are employed across conditions. The inherent design of a change detection task allows investigators to analyze separable period-specific patterns of activation across subjects which correspond to different cognitive behaviors such as the initial stimulus encoding and storage, maintenance of visual information while no relative information is displayed, and finally, the manipulation of a mental representation of visual information to match newly presented visual information before making a subsequent response about item change. Given our proposed task design, we hypothesize that observable differences corresponding to some aspect of each of these behaviors will be observable within the brain by analyzing task periods separately. In order to establish separate and differentiable neural profiles associated with each period, we combined conditions to determine similarities across conditions which are unique to task periods. Then, for the purpose of excluding incorrect trials, we directly contrast correct with incorrect trials for each task period to describe the temporal dynamics of a correct vs. an incorrect response. Finally, to determine differences between object and location memory for each task period, we contrasted these two conditions within each period.

## Materials and methods

### Subjects

Ten healthy volunteers (five women) 23–33 years old participated in the study. All subjects were in good health with no history of psychiatric or neurological disease and had normal or corrected-to-normal (with contact lenses) visual acuity. Subjects provided written informed consent. Study procedures were approved by the University of Texas Health Science Center at Houston Institutional Review Board.

### Task

The stimulus set included 9 colored squares (red, blue, green, yellow, orange, pink, purple, teal, and lime green) each subtended a visual angle of 1.3°. Stimuli were displayed on a black background in 6 of 16 possible locations. To prevent empty-space strategies during the location change condition, the location grid was created using two invisible concentric circles, each with eight possible locations equally spaced around the circle. During the pre-training phase, stimuli were displayed on a standard 17″ computer monitor (EIZO, Hakusan, Japan), and during the fMRI scans, stimuli were displayed on a screen that was mounted behind the subject's head, outside of the scanner. The image was reflected from a mirror onto a small screen directly above the subject's eyes for a viewing angle of 36.0°.

**Figure 1:**
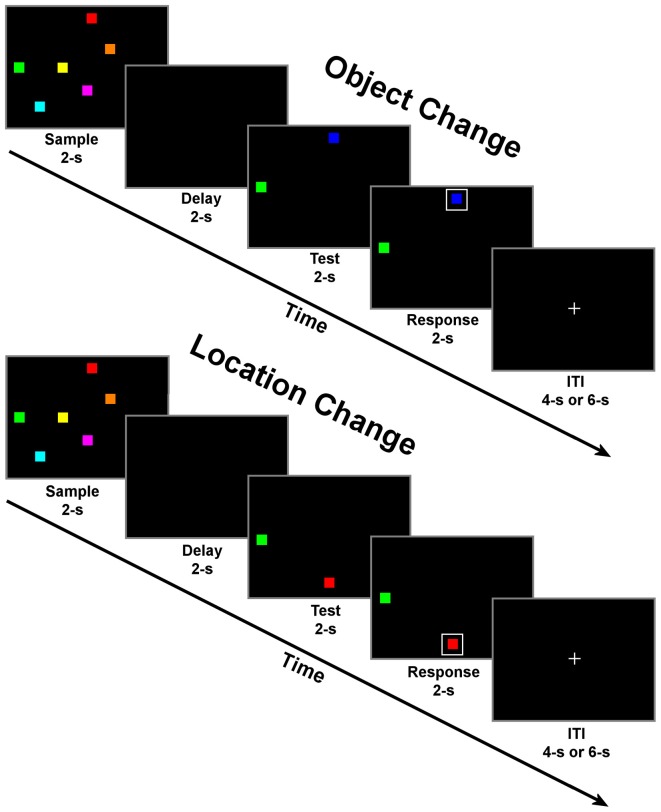
**Schematic of change detection task for object change (top) and location change conditions (bottom).** Each of the four task periods lasted for 2 s with a randomly selected inter-trial interval (ITI) of either 4 or 6 s. There were 23 trials of one condition per block for a total of 10 blocks with alternating conditions per block.

Subjects were tested in two memory conditions: change detection for objects and change detection for locations (Figure 1). Conditions were blocked and subjects were verbally informed about the condition prior to the start of each block. In both conditions, trials began with a 2 s fixation period. During the fixation period, a small white fixation cross (1.8° of visual angle) was presented in the middle of a black background. Following fixation, the sample period began. During this period, six colored squares were presented on the screen simultaneously for 2 s. Next, there was a 2 s delay period with an empty black display. The test period followed with two colored squares presented, one remained the same but one had changed in either color (object condition) or location (location condition) relative to the display presented during the sample. Subjects were instructed to covertly decide which square had changed in either color or location only, depending on the task condition. Finally, during the response period, a white box was randomly presented around one of the two colored squares (boxed item). Following the response period, the inter-trial interval (ITI) began, which consisted of a passive fixation display presented for a pseudo-randomized period of randomized 4 or 6-s. The time of this period was varied to induce a jitter across trials to prevent an overlap in the fMRI response curves, as implemented in previous fMRI studies (e.g., LoPresti et al., 2008; Brown, 2009; Ciaramelli et al., 2010). Additionally, the times associated with each task display (sample, delay, test, and response) were identical to allow for the study of task differences from one period to the next rather than focusing on only delay period activity. The relatively short periods (2 s) for each display in combination with the large number of trials (115) for each condition enables this task to be transferable to other neuroimaging techniques (such as MEG and EEG). The implementation of these task-specific parameters should provide novel insights into how the segregation of domain specific information progresses from encoding to maintenance and then a motor response in such a way that has not previously been described in the fMRI literature. The large number of trials in combination with identical stimulus displays of this task suggests that observed differences in brain activity are only associated with differences in the requirements of each task condition (memory of locations or identities).

Behavioral pre-training was conducted within 1 week prior to the fMRI scan to establish familiarity with the task and achieve an acceptable level of performance (>70%) for each subject. Several fMRI studies have reported changes in brain activity following training of working memory (Olesen et al., 2004; Westerberg and Klingberg, 2007), which would likely increase noise across subjects. A minimum performance criterion like that employed in this study should attenuate this variability. Pre-training consisted of a similar task to the one used in the fMRI scan and differed only by the response. During pre-training, subjects made a yes/no keyboard response to indicate whether or not the boxed item had changed. During the fMRI task subjects made a yes/no response using a response pad (Current Designs, Philadelphia, PA) to indicate whether or not the boxed item had changed. Subjects were not given feedback about whether or not they had made a correct response in either the pre-training or the fMRI task. Subjects completed 10 alternating blocks of 23 trials (five objects and five locations) for a total of 230 trials (higher than has previously been reported in the fMRI VWM literature) during both the pre-training session and the fMRI scan. All subjects were interviewed after each fMRI session to monitor task strategies. Questions about task strategy were asked such as the type of strategy used for each of the two conditions, if the strategy varied throughout the experiment, and if the strategy was the same one used during pre-training. Additional questions were presented in regards to the level of fatigue, comfortableness, possible confusion of different colors and/or the short distance used for object presentation in some trials.

A 5 min frontal-eye-field (FEF) localizer task was also used to identify the FEF region in each subject. This task consisted of five 30 s “on” and five 30 s “off” periods. During the “off” period, the subject was to stare at the central fixation cross and during the “on” period, the subject was informed to make a saccade to each of the four corners of the screen in a clockwise fashion and briefly fixate on the objects presented in each corner. The interview responses from pre-training suggested that some subjects may use a saccade-based configural strategy for the location condition but not for the object condition. However, activity in this FEF region did not vary significantly between the two task conditions across subjects suggesting that a similar saccade-based configural strategy was employed for each condition. Furthermore, self-reports from each subject identified the configural strategy as the primary strategy used for both conditions across a majority of the subjects.

### MRI acquisition protocol

MRI scans were acquired using a 3T Phillips (Bothell, WA) scanner located at the University of Texas Health Science Center at Houston. The scanner was equipped with an eight channel SENSE head coil. High resolution anatomical images were obtained using a magnetization-prepared 180° radio-frequency pulse and rapid gradient-echo (MP-RAGE) sequence. Sagittal slices were 1 mm thick and in-plane resolution was 0.938 × 0.938 mm. Functional images were acquired using a gradient recalled echo planar sequence that is sensitive to the blood-oxygen level-dependent (BOLD) signal. With this sequence, 33 axial slices were collected with a 2 s repetition time (TR), a 30 ms echo time (TE) and a flip angle of 90°. Voxel size was 2.75 × 2.75 × 3 mm. Each functional scan series consisted of 154 brain volumes. The first four volumes, collected before equilibrium magnetization was reached, were discarded resulting in 150 usable volumes. Following motion correction and slice timing correction, data were smoothed with a spatial Gaussian filter with root-mean-square deviation of 3 mm. Behavioral responses were collected using a fiber-optic button response pad (Current Designs).

### fMRI analysis

fMRI data analysis was performed using the Analysis of Functional NeuroImages software (AFNI) (Cox, 1996). Functional echo planar image (EPI) data were motion-corrected and aligned to individual anatomical data for each subject using the 3dAllineate plugin within AFNI. The fMRI response corresponding to each time point of correct trials was estimated using a multiple regression model using the AFNI function 3dDeconvolve. Using this model, eight box-car regressors corresponding to the onset of the four time periods for the two conditions were convolved with the gamma function. To correct for subject motion, six movement regressors were also included in the regression model. Importantly, the temporal jitter between trials in combination with limiting the analyses to correct trials provided accurate estimates of beta values for each trial period. The elimination of incorrect trials from our data analyses produced long 12–14 s gaps randomly where 20% (mean incorrect rate) of the trials had been located, which was excluded in the modeling of both baseline activity and task activity. Furthermore, the calculated regressors for each subject passed a high-threshold test of multicollinearity, which allowed for the estimation of activity corresponding to short task periods (2 s). This procedure for estimating short-period activity has been successfully employed by previous fMRI studies focusing on VWM that in some cases employed even shorter intervals than the 2 s we employed in this study (e.g., Pessoa and Ungerleider, 2004; Mohr et al., 2006; Sala and Courtney, 2007; Todd et al., 2011). The resulting beta coefficients for each voxel were transformed to Talairach space using auto_tlrc in AFNI.

#### Group analysis

The beta coefficients corresponding to each of the four task periods for correctly-answered trials for each condition were included in a voxel-wise 2-way within-subject ANOVA (AFNI program 3dANOVA3). In this ANOVA, subjects were treated as a random effect factor and condition and trial period were treated as fixed-effects factors. From this analysis, group-level activation maps were obtained for each of the four trial periods while contrasted with the fixation baseline. Group-level activation maps were also generated to contrast differences between the object and location change conditions during each of the four time periods. A second 2-way within-subjects ANOVA was performed for the correct vs. incorrect trial analysis which treated subjects as a random effect factor and trial type (correct or incorrect) and trial period as fixed-effects factors. Across both of these group analyses, a control was implemented to correct for multiple comparisons whereby a spatial cluster extent threshold was applied to the data using a Monte Carlo simulation (1000 randomizations) with an uncorrected voxel-wise threshold of *p* < 0.005. This calculation yielded a threshold of 12 contiguous voxels per cluster for a cluster significance of *p* < 0.05. As a result, only activation clusters above that threshold were reported. All results from the group analyses are projected on the inflated representation of the N27 brain.

## Results

### In-scanner task performance

Mean performance was not significantly different between the two conditions: 85.2% ± 6.8 for the object condition and 85.4% ± 6.5 for the location condition as determined by a paired samples *t*-test [*t*_(9)_ = 0.11, *p* < 0.92]. Mean response time was 882 ± 118 ms for the object condition and 851 ± 122 ms for the location condition and was not significantly different based on a paired samples *t*-test [*t*_(9)_ = 1.79, *p* < 0.11]. The mean response times of the correct and incorrect trials used in the correct vs. incorrect fMRI analyses were 853 ± 105 ms and 1030 ± 100 ms, respectively, and found to be significantly different using a paired samples *t*-test [*t*_(9)_ = 6.617, *p* < 0.0001].

### fMRI results

#### Combined object and location condition VWM

A goal of this study was to better understand VWM processes by analyzing each of its components: encoding (sample period), maintenance (delay period), retrieval/manipulation (test period). For the analysis of these components, we contrasted each of the two conditions (object and location) to baseline and combined them. Combined activation was analyzed separately for each task period to determine if the regression analysis was successful in teasing apart neural profiles which were expected for specific task periods such as a primary visual response during the sample period and a unilateral motor response for the response period. Previous fMRI research using change detection has shown different profiles of activation corresponding to a correctly or incorrectly detected change (Beck et al., 2001; Pessoa and Ungerleider, 2004). Therefore, only correct trials (a mean of 85% across subjects, 196 out of 230 trials across both conditions per subject) were used in this and further analyses. The duration of each period of the task was identical (2 s) to control for differences in activation associated with longer or shorter durations. Furthermore, the motor response was delayed by one period (2 s) from the onset of the test display.

**Figure 2:**
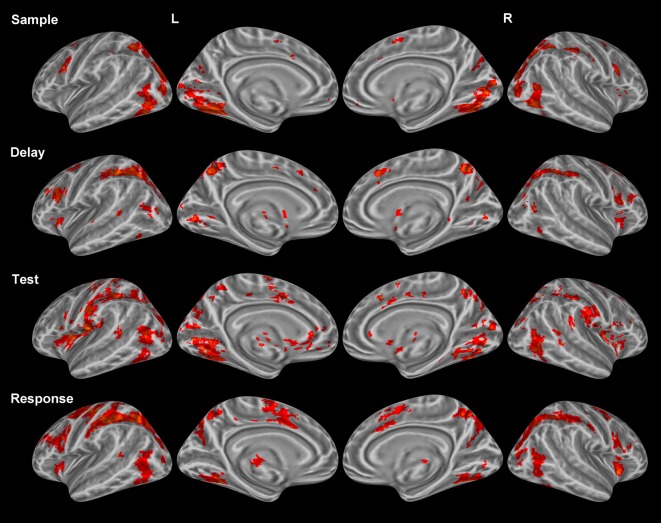
**Statistically significant (*p* < *0.05*, corrected for multiple comparisons) maps of activation combined across object and location conditions compared to baseline.** Top row: Sample period activity (initial 6-item display), second row: Delay period activity (blank screen), third row: Test period activity (2-item display), bottom row: Response period activity.

***Sample period.*** Visual encoding of both object and location information produced a cluster of activation extending from occipital regions to both temporal (ventral) and parietal (dorsal) regions (Figure 2). A large cluster of activation occurred in the inferior occipital, lingual, and fusiform gyri bilaterally—regions of the classically-defined ventral stream. This cluster of activation also extended to the classically-defined dorsal stream in the precuneus, cuneus, superior parietal lobule (SPL) bilaterally, and the inferior parietal lobule (IPL) in the right hemisphere. Clusters of activation were also observed in prefrontal regions during this period including a region (Brodmann area 9) of the classically defined dorsolateral prefrontal cortex (DLPFC), and the FEF in Brodmann area 6 (BA 6) (Table 1). Importantly, primary visual activation is observed during this time period only, which suggests that the regression model of analysis successfully teased apart the fMRI response associated with the sample display.

**Table 1:** **Significant activation clusters from combined conditions for each task period from the whole-head condition-period ANOVA**.

**Period**	**Brain region**	**BA**	**Hemisphere**	***x***	***y***	***z***	**No. of voxel**	***t*-value**
Sample	cuneus, lingual gyrus, fusiform gyrus, precuneus, superior parietal lobule, inferior/middle occipital gyrus		L + R	−29.0	53.0	−12.0	6687	9.115
	middle frontal gyrus	9	L	−43.0	−1.0	30.0	235	6.818
	middle frontal gyrus	9	R	45.0	−1.0	28.0	188	8.721
	inferior parietal lobule	40	R	45.0	31.0	44.0	170	5.173
	middle frontal gyrus	6	R	27.0	5.0	44.0	55	3.265
	precentral gyrus	4	L	53.0	17.0	38.0	39	3.792
	medial frontal gyrus	6	L + R	11.0	−5.0	50.0	37	3.597
	middle frontal gyrus	6	L	−29.0	1.0	48.0	32	2.877
Delay	inferior/superior parietal lobule, precuneus, cuneus, middle/superior occipital gyrus		L	−35.0	37.0	40.0	1829	12.253
	inferior/superior parietal lobule, precuneus, cuneus, middle/superior occipital gyrus		R	5.0	63.0	44.0	1576	12.203
	middle frontal gyrus	46	L	−41.0	−7.0	22.0	512	5.563
	lingual gyrus	18	L + R	−3.0	79.0	−4.0	310	5.593
	middle frontal gyrus	46	R	41.0	−21.0	20.0	294	5.580
	middle frontal gyrus	6	L	−37.0	1.0	42.0	291	4.222
	cingulate gyrus	32	L + R	9.0	−21.0	40.0	248	7.104
	insula	13	L	−27.0	−21.0	4.0	219	5.832
	caudate		L	−7.0	−1.0	−4.0	151	6.096
	insula	13	R	35.0	−15.0	8.0	150	4.670
	fusiform gyrus	37	L	−37.0	53.0	−10.0	140	5.465
	caudate		R	11.0	−7.0	−4.0	109	4.285
	middle frontal gyrus	6	R	27.0	1.0	56.0	107	4.336
	fusiform gyrus	37	R	39.0	61.0	−2.0	70	3.939
	superior frontal gyrus	8	R	27.0	−19.0	48.0	42	5.251
	thalamus		L	−5.0	13.0	6.0	40	4.699
Test	fusiform gyrus, lingual gyrus, inferior/middle occipital gyrus, cuneus, precuneus		L + R	−19.0	27.0	4.0	2276	7.645
	insula	13	L	−33.0	−1.0	14.0	940	9.519
	inferior parietal lobule	40	R	13.0	71.0	12.0	393	5.827
	caudate		R	19.0	−17.0	6.0	351	6.278
	thalamus		L	−19.0	27.0	4.0	241	12.239
	Insula	13	R	37.0	9.0	14.0	221	8.797
	postcentral gyrus	4	L	−61.0	17.0	28.0	189	5.435
	precentral gyrus	13	L	−39.0	21.0	56.0	168	6.382
	inferior parietal lobule	40	L	−33.0	41.0	40.0	152	6.028
	caudate		L	−19.0	71.0	50.0	102	6.377
	middle frontal gyrus	46	L	−41.0	−19.0	29.0	99	4.510
	precuneus	7	L	−11.0	−19.0	0.0	98	4.109
	parahippocampal gyrus	19	R	29.0	49.0	−6.0	62	4.439
Response	precentral gyrus, postcentral gyrus, inferior parietal lobule, supramarginal gyrus, precuneus, middle frontal gyrus	7	L	−41.0	35.0	36.0	5630	16.060
	inferior/superior parietal lobule, precuneus	7	R	29.0	51.0	42.0	3029	8.716
	inferior/middle occipital gyrus, fusiform gyrus, lingual gyrus	19	L	−37.0	71.0	−2.0	847	10.557
	medial frontal gyrus, cingulate gyrus	6	L + R	1.0	−1.0	48.0	451	6.278
	middle frontal gyrus	9	L	−41.0	−3.0	28.0	291	10.275
	insula	13	R	37.0	−19.0	−4.0	241	9.750
	middle frontal gyrus	9	R	53.0	−9.0	22.0	232	4.904
	inferior/middle occipital gyrus, fusiform gyrus, lingual gyrus	19	R	29.0	63.0	−12.0	221	7.012
	insula	13	L	−27.0	−23.0	2.0	168	6.452
	thalamus		L	−33.0	41.0	40.0	122	8.380

Activations are significant at a p < 0.05 corrected for multiple comparisons across both object and location conditions compared to baseline for each period of the task. L, left hemisphere; R, right hemisphere; L + R, a single cluster extending from one hemisphere to the other. Talaraich coordinates correspond to peak activation within the cluster.

***Delay period.*** The most widespread clusters of activation were observed in frontoparietal regions in similar locations reported above during the sample period (Figure 2). A shift in DLPFC activity was observed during this period from a slightly smaller and more posterior cluster occupying BA 9 to a larger and more anterior cluster of activation occupying BA 46. Similar to the sample period, a cluster of activation occurred in the precuneus, cuneus, middle occipital gyrus, and SPL bilaterally. This cluster of activation extended into the IPL and superior occipital gyrus bilaterally. Larger clusters of activation were observed bilaterally during this period when compared to the sample period in the FEFs (BA 6) (Table 1). Additionally, activation in primary visual regions was significantly reduced during this period as compared to the sample period which preceded it, which provides additional evidence to support task period separation based on the fMRI analysis performed.

***Test period.*** A single cluster of activation was observed in similar visual regions identified during the sample period (bilateral lingual, fusiform, precuneus, cuneus, inferior occipital, and middle occipital gyri). Additionally, clusters of activation were observed in the insula, caudate, and IPL bilaterally. In the right hemisphere, a single cluster of activation was observed in the parahippocampal gyrus (Figure 2).

***Response period.*** Primary motor response activation was observed in the left precentral and postcentral gyri (Figure 2) corresponding to a right index finger button press. This cluster of activation also occupied a portion of the left IPL, supramarginal gyrus, precuneus, and middle frontal gyrus (posterior). Two separate clusters of activation were observed in a region occupying the lingual, fusiform, middle occipital, and superior occipital gyri bilaterally. Bilateral clusters of activation covered the middle frontal gyrus near BA 9, the FEFs, and the insula. A large cluster of activation was observed in the right precuneus, IPL, and SPL and a small cluster was observed in the left thalamus. Finally, primary motor response activation was observed only during this task period and not during any other period.

#### Correct vs. incorrect trial analysis

**Figure 3:**
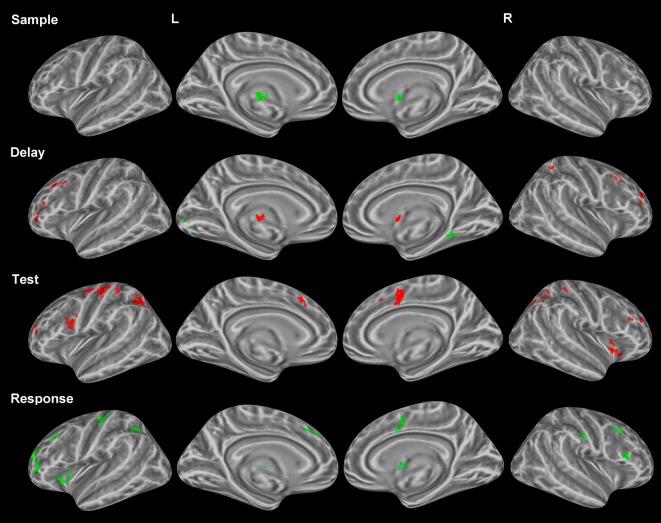
**Statistically significant (*p* < *0.05*, corrected for multiple comparisons) maps of activation contrasting correct trials (red) with incorrect trials (green) across both conditions for each task period.** Top row: Sample period activity (initial 6-item display), second row: Delay period activity (blank screen), third row: Test period activity (2-item display), bottom row: Response period activity.

As mentioned earlier, several studies have identified unique profiles of activation for both correctly and incorrectly identified changes during change detection. Therefore, only correct trials were used in the above analyses. We compared correctly and incorrectly responded trials collapsed across object and location change conditions yielding differential neural profiles associated with each. *Sample period*. Incorrect trial activation was observed in the left thalamus and the right putamen (Figure 3). No other clusters of activation were observed during the sample period.

***Delay period.*** Activation for incorrect trials was observed in the left lingual gyrus and the right parahippocampal gyrus (Figure 3). Clusters of activation for correct trials during this period were observed in various regions including several bilateral prefrontal regions covering the middle frontal gyrus in BA 10 and the FEFs. Additionally, correct trial clusters were observed in the right putamen, the left thalamus, and the right IPL (Table 2).

**Table 2:** **Significant activation clusters of correct vs. incorrect trial contrast from whole-head trial type-period ANOVA**.

**Period**	**Trial type**	**Brain region**	**BA**	**Hemisphere**	***x***	***y***	***z***	**No. of voxel**	***t*-value**
Sample	Incorrect	thalamus	92	L	−11.0	15.0	6.0	92	4.289
		putamen	68	R	13.0	−5.0	0.0	68	5.105
Delay	Correct	middle frontal gyrus	10	R	33.0	−37.0	18.0	117	5.720
		putamen		R	15.0	1.0	4.0	94	7.607
		middle frontal gyrus	6	R	45.0	−11.0	48.0	88	4.260
		middle frontal gyrus	10	L	−31.0	−43.0	2.0	83	4.109
		inferior parietal lobule	40	R	39.0	37.0	56.0	69	5.118
		thalamus		L	−3.0	19.0	2.0	67	6.734
		middle frontal gyrus	6	L	−27.0	−1.0	48.0	60	5.611
	Incorrect	lingual gyrus	17	L	−11.0	91.0	0.0	164	4.152
		parahippocampal gyrus	37	R	27.0	43.0	−8.0	97	4.830
Test	Correct	precentral/postcentral gyrus	4	L	−31.0	29.0	56.0	819	9.461
		cingulate gyrus	24	L + R	9.0	−1.0	46.0	473	6.873
		precuneus, inferior parietal lobule	7	L	−29.0	59.0	42.0	334	5.781
		precuneus, inferior parietal lobule	7	R	11.0	65.0	24.0	323	6.211
		middle frontal gyrus	46	L	−49.0	−19.0	30.0	193	6.101
		insula	13	R	39.0	−19.0	0.0	157	9.107
		postcentral gyrus	3	R	39.0	33.0	54.0	94	5.002
		middle frontal gyrus	10	L	−31.0	−45.0	18.0	77	4.261
		middle frontal gyrus	9	R	47.0	−13.0	46.0	71	4.844
Response	Incorrect	cingulate gyrus	24	L + R	−3.0	−11.0	42.0	365	5.566
		middle frontal gyrus	10	L	−27.0	−47.0	16.0	360	5.105
		middle frontal gyrus	6	R	47.0	−11.0	46.0	279	5.023
		putamen		R	15.0	−3.0	10.0	269	7.200
		middle frontal gyrus	9	L	−41.0	−31.0	30.0	184	4.373
		precentral/postcentral gyrus	4	L	−21.0	23.0	52.0	154	4.533
		insula	13	L	−35.0	−11.0	8.0	148	8.999
		middle frontal gyrus	46	R	47.0	−23.0	16.0	92	4.641
		inferior parietal lobule	40	L	−33.0	51.0	38.0	85	4.959
		thalamus		L	−11.0	21.0	8.0	81	7.243
		postcentral gyrus	3	R	51.0	17.0	32.0	78	4.820

Activation clusters are significant at a p < 0.05 corrected for multiple comparisons contrasting correct and incorrect trials for each period of the task. Activation differences between trial type are identified as significantly active for that trial type when compared to baseline. L, left hemisphere; R, right hemisphere; L + R, a single cluster extending from one hemisphere to the other. Talaraich coordinates correspond to peak activation.

***Test period.*** Correct trial clusters were observed in the bilateral precuneus, IPL, DLPFC (BA 46 and BA 9), cingulate gyri (Figure 3), BA 10, and the right insula. Additionally, clusters were found in the left precentral/postcentral gyrus and right postcentral gyrus suggesting preparation for a motor response.

***Response period.*** Incorrect trial clusters were observed in similar regions to those identified during the test period for correct trials. Activation over the bilateral DLPFC (BA 46 and BA 9) as well as the left precentral/postcentral gyrus, left BA 10, bilateral cingulate gyri, and the right postcentral gyrus were observed (Table 2).

#### Object vs. location

Each of the components of VWM was explored in the context of domain specificity such that both object and location conditions were contrasted directly across each trial period. All sample and test displays presented during the object condition were also presented during the location condition. The resulting differences between conditions occurred within a broad range of areas across all cortical lobes and even some subcortical structures. This analysis was performed to tease apart period-specific differences across object and location memory conditions to address the issue of domain specificity.

**Figure 4:**
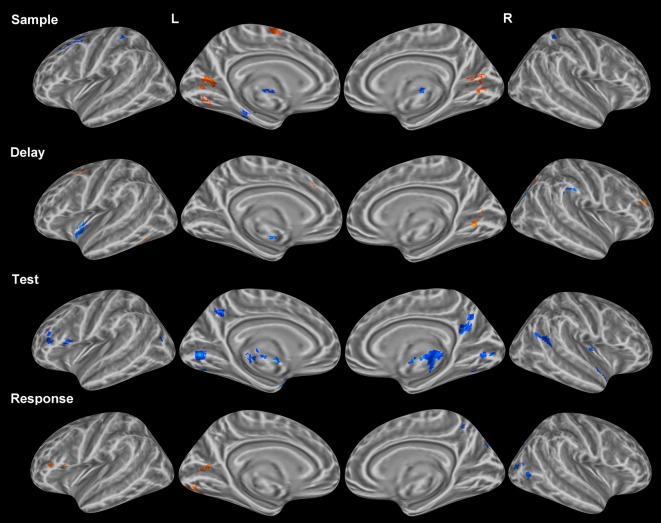
**Statistically significant (*p* < *0.05*, corrected for multiple comparisons) maps of activation contrasting object change condition (orange) and location change condition (blue) for each task period.** Top row: Sample period activity (initial 6-item display), second row: Delay period activity (blank screen), third row: Test period activity (2-item display), bottom row: Response period activity.

***Sample period.*** Bilateral clusters of activation were observed in the lingual gyri (ventral stream) for the object condition when compared to the location condition (Figure 4). Bilateral clusters of activation were also observed in the IPL (dorsal stream) for the location condition. Additionally, clusters of activation were observed in the red nucleus (bilaterally) and the left FEF for the location condition. A cluster of activation in a region occupying left BA 6, an area classically defined as the supplementary motor area (SMA), corresponded to the object condition.

***Delay period.*** Clusters of activation covered various regions for the object condition and the location condition (Table 3). Object clusters were identified in the left FEF (BA 6) and the right SPL. Location clusters were found in the left insula (BA 13), left putamen, and right IPL (Figure 4).

**Table 3:** **Significant activation clusters of object vs. location contrast from whole-brain condition-period ANOVA**.

**Period**	**Condition**	**Brain region**	**BA**	**Hemisphere**	***x***	***y***	***z***	**No. of voxel**	***t*-value**
Sample	Object	lingual gyrus	18	R	25.0	67.0	12.0	180	4.047
		lingual gyrus	18	L	−11.0	75.0	4.0	117	5.954
		medial frontal gyrus	6	L	−5.0	7.0	60.0	80	4.310
		lingual gyrus	18	L	−17.0	71.0	−10.0	71	3.486
	Location	middle frontal gyrus	6	L	−25.0	−17.0	36.0	258	6.495
		red nucleus		L + R	1.0	21.0	−4.0	145	10.583
		inferior parietal lobule	40	R	37.0	41.0	56.0	74	3.560
		inferior parietal lobule	40	L	−37.0	41.0	56.0	54	3.061
		fusiform gyrus	37	L	−53.0	45.0	−12.0	52	5.919
Delay	Object	lingual gyrus	18	R	17.0	67.0	6.0	231	5.780
		middle frontal gyrus	9	R	37.0	43.0	24.0	99	4.153
		middle frontal gyrus	6	L	−25.0	1.0	54.0	71	4.332
		superior parietal lobule	7	R	25.0	61.0	40.0	52	4.640
		cingulate gyrus	32	L	−3.0	−15.0	38.0	50	3.484
	Location	insula	13	L	−43.0	3.0	2.0	208	5.632
		putamen		L	−23.0	−9.0	−2.0	108	4.890
		middle occipital gyrus	19	R	37.0	77.0	18.0	101	4.982
		inferior parietal lobule	40	R	53.0	33.0	42.0	89	4.619
Test	Location	lingual gyrus	18	L + R	−5.0	77.0	4.0	630	6.295
		red nucleus		L + R	−11.0	47.0	−6.0	597	9.992
		retrospinal cortex	29	L + R	9.0	47.0	8.0	553	6.050
		putamen, thalamus		L + R	21.0	5.0	−2.0	416	8.000
		inferior frontal gyrus	44	L	−35.0	−31.0	12.0	230	5.879
		temporal pole	38	R	45.0	−11.0	−18.0	109	4.324
		insula	13	R	33.0	19.0	14.0	98	7.064
		superior temporal gyrus	22	R	41.0	45.0	16.0	68	4.632
		middle occipital gyrus	19	L	−33.0	77.0	20.0	61	5.176
		Parahippocampal gyrus, uncus	38	L	−33.0	−3.0	−16.0	59	6.902
Response	Object	fusiform gyrus, lingual gyrus	18	L	−23.0	81.0	−4.0	76	5.320
		inferior frontal gyrus	45	L	−49.0	−27.0	10.0	75	6.497
	Location	precuneus, inferior parietal lobule	31	R	13.0	53.0	46.0	231	4.962
		middle occipital gyrus	19	R	39.0	67.0	6.0	173	6.334

Activations are significant at a p < 0.05 corrected for multiple comparisons contrasting the object and location conditions for each period of the task. Activation differences between conditions are identified as significantly active for that condition when compared to baseline. L, left hemisphere; R, right hemisphere; L + R, a single cluster extending from one hemisphere to the other. Talaraich coordinates correspond to peak activation.

***Test period.*** Clusters of activation were observed only for the location condition and found in the posterior cingulate gyri and putamen, bilaterally. (Table 3). Additional unilateral location clusters were identified in the right insula, the left anterior parahippocampal gyrus, and a region in the inferior frontal gyrus classically considered part of Broca's area (BA 44).

#### Baseline suppression

**Figure 5:**
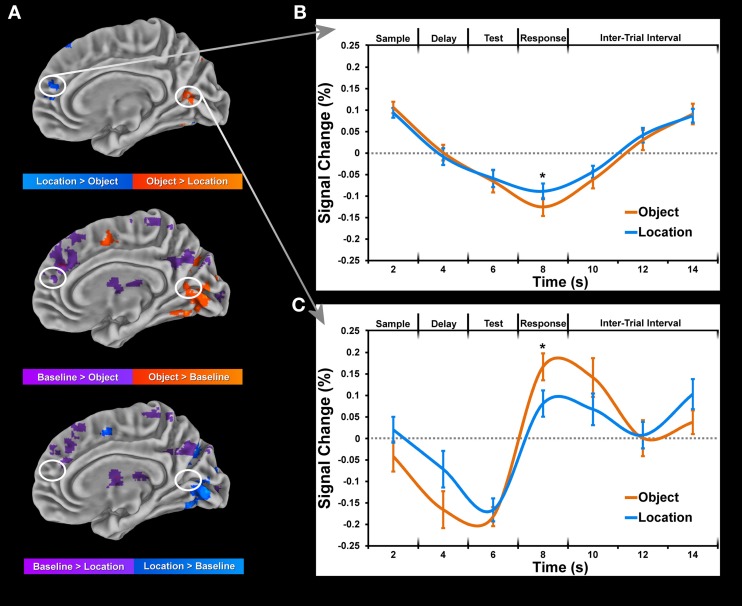
**Data is normalized for each block by computing percent change from baseline for each condition and subject.** This data was then submitted to a deconvolution algorithm (3dDeconvolve) which estimated the corresponding time-courses of activation using a tent function to model the sample display onset for each condition separately. Only trials for which the subject made a correct response were included in the deconvolution. **(A)** Maps of significant (*p* < *0.05*, corrected for multiple comparisons) activation during sample period between object and location conditions (top) and each condition to baseline (bottom 2 rows). **(B)** Time-course of selected cluster of activation within the right lingual gyrus showing greater location than object activation as a result of baseline suppression. **(C)** Time-course of selected cluster of activation within the right medial frontal gyrus showing greater object than location activation as a result of greater object than baseline activation. Error bars represent standard errors of the mean. Asterisks (^*^) denote significant difference for paired *t*-test (*p* < *0.001*, corrected for multiple comparisons).

Each condition was compared to baseline separately for each task period, paying particular attention to coordinates of peak activation observed in the cluster analysis of conditions. In many instances, greater location than object activity was not observed when each condition was compared to baseline directly. Moreover, baseline activity in the object condition often coincided with weak or no activity in the location condition at the same coordinates (Figure 5A). This finding suggests a suppression of baseline activity during the object condition and less (if any in some cases) suppression of baseline activity during the location condition (Figure 5B). Only differences between conditions which were identified as being significantly active when compared to baseline (Figure 5C) are reported in Tables 2, 3. Consequently, all clusters of activation which represented baseline suppression (Figure 5B) were removed from the analysis to provide a clearer profile of the activation of each condition.

## Discussion

The resulting analyses of this study of object and location memory identified unique brain patterns associated with location and object memory in regions extending across the entire brain during each of the four task periods. Furthermore, dissociable patterns of brain activity were observed in frontal, parietal, and temporal cortices, which evolved in amplitude and location as the task progressed from sample through to response displays. The task used in this study was unique in that a larger sample of trials (196 per subject on average) was used than has previously been employed in the fMRI literature focusing on VWM and the change detection paradigm controlled for equal duration among task periods. Furthermore, we compared object and location VWM with identical stimuli and stimuli displays (sample period and test period) across object and location conditions. To the best of our knowledge, an experiment incorporating these important controls has never before been described in the fMRI literature. Previous fMRI studies have identified differences between encoding, maintenance, and retrieval/manipulation across various VWM tasks including change detection, N-back, and Sternberg tasks (Manoach et al., 2003; Ranganath et al., 2004, 2005; Mohr et al., 2006; Woodward et al., 2006; Hester et al., 2007). However, none of these studies have compared distinct task periods using equal duration across periods or in combination with such a large number of trials and identical displays as described here.

### Period-specific activations

Given our task design, we were able to deconstruct each trial into 4 distinct periods (sample, delay, test, response) across subjects as evident in the differential patterns of activation associated with each period (Figure 2). While each task period lasted for 2 s, some previous fMRI research has argued in favor of partial trials and differential time periods across each task period to more effectively tease apart period-specific activations (e.g., Serences, 2004; Amaro and Barker, 2006). However, this study focused on activity specific to each task period, which made it impractical to jitter periods while providing a sufficient number of jitter combinations across trials and conditions. Furthermore, previous studies have shown differential BOLD activations associated with longer delay periods for VWM tasks (e.g., Elliott and Dolan, 1999; Schluppeck et al., 2006).

#### Frontoparietal network

Activation of the frontoparietal network was observed during the earliest stage of the task (sample period), suggesting that executive function in conjunction with the storage of visual information occurs while the information is first encoded. Previous fMRI studies have consistently shown activation bilaterally in the frontparietal network (e.g., Miller and Cohen, 2001; Curtis and D'Esposito, 2003; Leung and Alain, 2011) as we have shown here. During the sample period, the subject's goal was to encode and store observed visual information in a meaningful and efficient way. In contrast, during the delay period the subject's mental resources were working to keep the visually displayed information in memory which is supported by communication among prefrontal and parietal structures. This latter result concerning frontal and parietal structures is consistent with recent evidence of a central-executive resting state network consisting of correlated activity in DLPFC and parietal lobe structures (Damoiseaux et al., 2006; Seeley et al., 2007). Furthermore, studies have reported this network (the specific frontal and parietal regions) to be active during the maintenance and manipulation of information (Miller and Cohen, 2001; Petrides, 2005; Müller and Knight, 2006). Although some studies have described frontoparietal activation during a sample period (e.g., Curtis and D'Esposito, 2003), the sample period incorporated in the study described here is longer than the typical sample duration reported in the literature (e.g., less than 1 s). We found that during the sample period, activation in the classically-defined DLPFC (BA9 and 46) was localized to a more posterior and dorsal region occupying BA 9, but later shifted to a more anterior region of DLPFC in BA 46 during the delay period. This shift may correspond to divergent roles of sub-regions of the DLPFC which has been characterized as the center for goal-directed behavior (Miller and Cohen, 2001; Stuss and Levine, 2002). Concerning parietal structures, we showed activity in the right IPL during the sample period and the delay period but the left IPL was only shown to be active during the delay. This may suggest disparate hemispheric roles of the IPL not previously described in the VWM literature, perhaps because encoding and maintenance periods are often combined. The addition of left IPL activity during the delay period could reflect a temporal dynamic of the IPL associated with rehearsal of visual information after it is encoded.

#### Visual network

Regions of primary visual cortex in the occipital lobe as well as dorsal and ventral extrastriate regions were shown here to be active during the sample period when six colored squares were presented, which is in agreement with some previous VWM studies employing simple visual displays similar to this one (e.g., Manoach et al., 2003; Koshino et al., 2005). Interestingly, some of these same visual regions were active during the delay period in the absence of a visual stimulus. We believe this may reflect a strategy often reported by subjects during the post-fMRI interview in which the after-image for the configuration of sample items was actively maintained for comparison to the test display.

#### Subcortical activation

Subcortical structures were active throughout all task periods except the sample period, which is in agreement with subcortical findings from previous fMRI studies focusing on VWM tasks (e.g., Levitt et al., 2002; Lewis et al., 2004). The head and body of the caudate were active bilaterally during both the delay and test periods suggesting a role in maintenance and retrieval of specific visual information. Indeed, the caudate has been implicated in both learning (Delgado et al., 2005; Seger and Cincotta, 2005) and working memory (Manoach et al., 2000; Levitt et al., 2002; Lewis et al., 2004), which may help explain this structure's involvement in this VWM task. Furthermore, recent evidence suggests the head of the caudate is a region responsible for planning (Seger and Cincotta, 2005), likely due to its strong connections with prefrontal regions (Frančois-Brosseau et al., 2009; Jankowski et al., 2009; Provost et al., 2010). In light of these findings, the caudate is likely involved in maintenance of information during the delay period and motor planning in the test period.

Activation was observed in the left thalamus throughout the task (delay, test, and response) and was often the most statistically significant cluster of activation (*t* range 4.7–12.2). The focus of activation within the thalamus moved from the dorsomedial nucleus during the delay period to the medial pulvinar nucleus during the test and response periods. A recent diffusion tensor imaging (DTI) study by Piras et al. (2010) found a negative relationship between bilateral thalami mean diffusivity and WM performance among neurologically intact individuals. The left dorsomedial nucleus in particular has been shown to play a role in reading and verbal memory tasks (Speedie and Heilman, 1982; Sandson et al., 1991; Johnson and Ojemann, 2000). In regards to the pulvinar, several studies have shown increased pulvinar activation in the context of visual search and VWM tasks (Soto et al., 2007; Grecucci et al., 2010; Rotshtein et al., 2011).

### Domain-specificity (object vs. location)

For comparison of object and location conditions, we initially found substantially more location clusters of activation across all periods. In order to assess the role of baseline suppression, we contrasted each condition with baseline activity and in some instances (Figure 5) and identified stronger or weaker baseline suppression between object and location conditions within the same set of voxels. Many of the regions corresponding to baseline suppression during the object condition have been identified previously in the fMRI literature as part of the default mode network (DMN) which is typically deactivated during a task. To the best of our knowledge, this distinction of differences between conditions (baseline suppression or activation greater than baseline) has not been made in previous fMRI studies looking at WM, which may distort findings comparing location and object memory. For example, to claim that activation in medial prefrontal areas (Figure 5A) is associated with location memory and not object memory would be inaccurate since that same region is significantly active during fixation (baseline activity) and is only more inhibited during object memory (Figure 5B). For example, a recent study by Leung and Alain (2011) reported several negative values for an object change condition from an experiment on object and location auditory WM. While this finding suggests greater baseline than object activity, the investigators did not distinguish between this type of difference and all others in the study. Therefore, we have taken into account the change in activity relative to baseline to properly tease apart domain-specific brain regions of the sort reported in this study.

#### Dorsal-ventral separation

Contrary to some of the previous fMRI studies, we did not find a dorsal-ventral separation in the prefrontal cortex corresponding to location and object memory, respectively. Following the discovery of a visual dorsal-ventral separation in non-human primates by Ungerleider and Mishkin (1982), many investigators explored this dissociation using positron emission tomography (PET) and fMRI in humans. The focus of those studies eventually led to non-visual regions including prefrontal areas. An early review of the WM literature focusing on the DLPFC by Levy and Goldman-Rakic (2000) reviewed the studies which claimed to show domain-specific differences within the prefrontal cortex corresponding to the maintenance of spatial and object information. While no definite conclusions were drawn, it was suggested that each domain (object and location) was separable within the prefrontal cortex. Several recent fMRI experiments studying WM have focused their analysis on dorsal and ventral prefrontal regions in an attempt to identify such a spatial-object dissociation, respectively, with varying results across each study showing evidence either in favor of or against a prefrontal dorsal-ventral separation (Sala et al., 2003; Rämä et al., 2004; Mohr et al., 2006; Sala and Courtney, 2007; Volle et al., 2008). The statistical strength resulting from our dataset (>100 trials per condition) should have been sufficient to detect any dorsal-ventral separation in prefrontal regions. While no prefrontal separation was observed, a dorsal-ventral separation during the sample period was identified in visual regions as expected (e.g., Ungerleider and Mishkin, 1982). In particular, object activation was found in the lingual gyrus bilaterally while location activation was associated with the IPL bilaterally during this period. This finding is in agreement with some of the previous VWM studies which reported a visual dorsal-ventral separation (Sala et al., 2003; Borowsky et al., 2005; Mohr et al., 2006). This type of dorsal-ventral separation was not observed in any other region or task period. Importantly, this visual separation was not found during the test period when two items were presented, suggesting the regions involved in processing the object and location information did not significantly differ.

#### Domain-specific encoding and maintenance

Domain-specific profiles of activation were observed separately for both the sample and delay periods. During the sample period, a location-domain profile of activation consisted of the IPL bilaterally, the left medial frontal gyrus and the red nucleus bilaterally while an object-domain profile consisted of activation in the lingual gyrus bilaterally and the left SMA. Previous fMRI studies on WM have identified location activation during the delay period in the IPL (Sala et al., 2003; Mohr et al., 2006; Sala and Courtney, 2007; Leung and Alain, 2011) and left medial frontal gyrus (Mohr et al., 2006; Sala and Courtney, 2007; Harrison et al., 2010; Leung and Alain, 2011). Previous studies have also reported on object activation in the lingual gyrus (Sala et al., 2003; Mohr et al., 2006) and the left SMA (Sala et al., 2003; Leung and Alain, 2011) during maintenance. The finding of activation in the red nucleus bilaterally associated with location memory has not previously been reported, to the best of our knowledge. Furthermore, activation in this region was shown to be of greatest statistical significance (*t* > 10.5) compared to all other regions resulting from the condition contrast. The red nucleus is classically defined as part of the rubrospinal tract and is involved in motor coordination. While the change detection task used in this experiment required motor coordination (single button-press) at the end of each trial, the amount of motor involvement was unlikely to differ for each condition. Moreover, red nucleus activation was observed during the earliest point of the trial (sample period) rather than during the end of the trial corresponding to the response period. Perhaps location activation in this region reflects a spatial organization similar to that required for motor coordination, which is not necessary during the object condition. Further investigation of this structure in the context of WM is required in order to better assess its role in location memory.

During the delay period, the location-domain activity occurred in the left insula and the right IPL while the object-domain activity occurred in the right SPL and left medial frontal gyrus. Activation in the IPL during the maintenance of location information has been shown in various WM studies; however, insula activation for location memory has not been demonstrated. Several studies have implicated the insula in mental navigation tasks (Ghaem et al., 1997; Campbell et al., 2009) which is likely to involve spatial representation as well. Recently, a functional connection between the insula and the anterior cingulate has been identified and described as a resting state network corresponding to the salience system (Taylor et al., 2009). Saliency in the context of maintenance of location information may describe a strategy in which a salient configuration is identified within an ambiguous spatial configuration. Indeed, this is in line with the strategy which subjects often reported for the location condition of this task. Object activation was also observed in the right parietal lobe, in a more medial region (SPL) compared to the location activation observed nearby (IPL). Several studies have identified a location-object dissociation within the IPS (Xu and Chun, 2006; Harrison et al., 2010), however, the dissociations pertained to either condition-specific load differences or a superior-inferior separation. The separation of object and location information in medial and lateral parietal regions (or SPL and IPL), respectively, may reflect a domain-specific dissociation along a different axis than has been previously identified. The object activation observed in the left medial frontal gyrus exists in the same location which was previously occupied by location activation during the sample. It is unclear why similar activation is observed across both conditions spanning different task periods, but this overlap may relate to a difference in the strategies used for each condition rather than a difference in the storage of domain-specific information.

#### Retrieval

The contrast of location vs. object memory conditions yielded greater location than object activations throughout cortical and subcortical regions. We believe these differences reflect divergent retrieval of visual information corresponding to distinct strategies for each condition. Post-fMRI interviews with subjects identified a specific strategy for the location condition in which subjects created a mental configuration of the six colored squares during the sample period and attempted to fit the two new squares during the test period into the mental representation to determine which item had changed location. Location activation during this period was observed in several key regions including the retrosplenial cortex (BA 29), the anterior parahippocampal gyrus (BA 38), the putamen of the basal ganglia, the inferior frontal gyrus (BA 44), and the right insula cortex. Previous fMRI studies have described the retrosplenial cortex's role in the processing of and memory for visuospatial orientation (Kataoka et al., 2006; Vogt et al., 2006; Antal et al., 2008), while the anterior parahippocampus has been implicated in the encoding (Buffalo et al., 2006; Xu et al., 2010) and retrieval (Gabrieli et al., 1997) of spatial information. Interestingly, a recent VWM study by Schon et al. (2009) identified the left anterior hippocampal gyrus and the retrosplenial cortex as regions with increased activation during retrieval periods when larger memory load was required. Since subjects performed similarly across conditions and an equal number of items (6) were presented throughout, this suggests disparate hippocampal activity in the absence of memory load differences. Furthermore, subcortical activation in the basal ganglia has been reported in studies focusing on parity judgments in delayed mental rotation tasks (Alivisatos and Petrides, 1997; Harris et al., 2002; Crucian et al., 2003). A recent study combining fMRI and DTI by Umarova et al. (2010) identified a functional network of regions corresponding to visuospatial attention consisting of BA 44, the insula cortex, the putamen, and the medial frontal gyri. This network appears to coincide with the location network of regions activated during the retrieval period of this task.

#### Maintenance is key

Activation of a specific group of regions during the delay period was involved with the correct identification of the changed item. These regions included bilateral FEFs, the putamen, and the right IPL. Few differences appeared between correct and incorrect trials during encoding, suggesting that subjects were attending to the visual display equally at the beginning of the trial regardless of the outcome. Activation in the right posterior parahippocampal gyrus (BA 37) coincided with incorrect trials during the delay period which may have been a result of proactive interference from previous trials. Furthermore, a study by Pessoa et al. (2002) identified a contingency between increased activation in the IPS and FEF regions and increased performance during the delay. Additionally, the right IPL has previously been shown to play a role in correct responses during the delay period based on a functional connectivity analysis of right IPL activation and intermediate-tier regions (Bressler et al., 2008). The finding of activation in the putamen during this period is in line with several reviews purporting the basal ganglia's role in learning and memory (Packard and Knowlton, 2002; Grahn et al., 2009; Baier et al., 2010).

The correct decision was made during the test period, thus correct-trial activation enabled the subject to correctly identify the changed item during the test rather than the response period (Figure 3). Activation in the IPL bilaterally, the left DLPFC, the right insula, the SMA bilaterally, and left motor regions comprised a profile of activation which coincided with the correct identification of the item which had changed. Conversely, a similar profile of activation was observed during the response period for incorrect trials, suggesting that subjects attempted to resolve the detection of change, but too late. Possibly incomplete maintenance of displayed information resulted in delayed retrieval or weak-confidence guesses evinced by the differential activation described during the delay period.

### Limitations

Based on our task design and the subsequent analyses, there are several limitations of our results. While, the deconvolution method utilized in this study produced differentiable period-specific activations (i.e., visual pathway activations during the sample display and left motor cortex during the response period), the use of partial trials or variable task periods (e.g., Serences, 2004; Amaro and Barker, 2006) may have produced slightly more distinct period activations. Furthermore, given more trials, it would have been possible to test for different task period lengths while counterbalancing for all possible combinations across trials.

## Concluding remarks

In summary, our results demonstrate that VWM is functionally separable into domain-specific components. The combination of controls described and implemented in this study have not been previously reported in the fMRI literature and provide novel insight into the neural profiles corresponding to location and object memory as they relate to one another in the absence of any task difference. The correct vs. incorrect analysis suggests that the delay period is the critical point during the trial to correctly identify a changed item. Accordingly, there is an emphasis on the functional differences observed during the maintenance of visual information in the object domain as compared to the location domain. In particular, activity in right SPL and MFG regions bilaterally corresponds to object memory while activity in right IPL, left insula, and right middle occipital gyrus corresponds to location memory. These findings suggest that object memory may more generally be associated with rostral brain structures while location memory may be associated with caudal structures.

### Conflict of interest statement

The authors declare that the research was conducted in the absence of any commercial or financial relationships that could be construed as a potential conflict of interest.
